# Cannabidiol and Other Cannabinoids in Demyelinating Diseases

**DOI:** 10.3390/ijms22062992

**Published:** 2021-03-15

**Authors:** Carmen Navarrete, Adela García-Martín, Alain Rolland, Jim DeMesa, Eduardo Muñoz

**Affiliations:** 1 Emerald Health Pharmaceuticals, San Diego, CA 92121, USA; cnavarrete@emeraldpharma.life (C.N.); adelagarcia@emeraldpharma.life (A.G.-M.); arolland@emeraldpharma.life (A.R.); jimdemesa@emeraldpharma.life (J.D.); 2 Instituto Maimónides de Investigación Biomédica de Córdoba, 14004 Córdoba, Spain; 3 Departamento de Biología Celular, Fisiología e Inmunología, Universidad de Córdoba, 14071 Córdoba, Spain; 4 Hospital Universitario Reina Sofía, 14004 Córdoba, Spain

**Keywords:** cannabinoids, cannabidiol derivatives, demyelinating diseases

## Abstract

A growing body of preclinical evidence indicates that certain cannabinoids, including cannabidiol (CBD) and synthetic derivatives, may play a role in the myelinating processes and are promising small molecules to be developed as drug candidates for management of demyelinating diseases such as multiple sclerosis (MS), stroke and traumatic brain injury (TBI), which are three of the most prevalent demyelinating disorders. Thanks to the properties described for CBD and its interesting profile in humans, both the phytocannabinoid and derivatives could be considered as potential candidates for clinical use. In this review we will summarize current advances in the use of CBD and other cannabinoids as future potential treatments. While new research is accelerating the process for the generation of novel drug candidates and identification of druggable targets, the collaboration of key players such as basic researchers, clinicians and pharmaceutical companies is required to bring novel therapies to the patients.

## 1. Introduction

*Cannabis sativa*, which contains about 545 natural compounds of different chemical structures known as cannabinoids, and its use for medicinal purposes, is centuries old [1]. The contemporary history of use of medical cannabis begins in the 19th century when an Irish physician, William Brooke O’Shaughnessy, introduced the cannabis plant into Western medicine for its analgesic, anti-inflammatory and anticonvulsant properties [2]. The most advanced characterization of different compounds extracted from the cannabis plant, termed phytocannabinoids, was undertaken during 1960s by the Israeli researcher Dr. Ralph Mechoulam, whose group isolated and reported among others on the chiral cannabidiol [3] and the psychotropic Δ9-tetrahydrocannabinol (Δ9-THC), two of the main bioactive compounds in the plant [4,5].

In 1985, the Food and Drug Administration (FDA) approved the first two cannabinoid derivatives for clinical use named dronabinol and nabilone. Dronabinol contains the trans isomer of Δ9-THC (synthetically derived) dosed in a gelatin capsule. This drug was approved for two indications: 1) chemotherapy-induced nausea and vomiting; and 2) anorexia in acquired immunodeficiency syndrome (AIDS) patients [6]. The second, nabilone, is a synthetic cannabinoid that mimics the activity of Δ9-THC. This drug was approved by the FDA to treat chemotherapy-induced nausea [7]. Both drugs are available only as oral capsules. In 2005, the authorization of Sativex, a mixture of Δ9-THC and CBD indicated to treat pain and spasticity in MS, supposed a milestone in cannabinoids research [7,8]. Furthermore, cannabidiol oral solution named Epidiolex, which presents beneficial effects for treatment of severe childhood epilepsy, has been recently approved by the FDA as a non-controlled substance.

Considerable interest in CBD is emerging due to its beneficial antiepileptic [8], neuroprotective in hypoxia-ischemia [9], anxiolytic, antipsychotic [10], anti-inflammatory [11] and anticancer properties [12], among others (Table 1). In the past, CBD has received less attention as a potential drug candidate than Δ9-THC although it has been commonly used in cannabis-based formulations.

**Table 1:** Therapeutic potential of CBD, analogs, and derivatives.

**Compounds**	**Therapeutic Potential**	**References**
CBD	Inflammation	Mecha et al., 2012 [13]
	Epilepsy	Burstein et al., 2015 [11]
	Cancer	Mori et al., 2017 [9]
	Anxiety	Kis et al., 2019 [12]
	Neuroprotection	García-Gutiérrez et al., 2020 [10]
	Myelination	Li et al., 2020 [8]
CBDA	Inflammation	Pellati et al., 2018 [14]
	Cancer	
	Antimicrobial	
CBDVA-C3	Convulsion	Anderson et al., 2019 [15]
CBDV	Convulsion	Zamberletti et al., 2019 [16]
	Epilepsy	Morano et al., 2020 [17]
	Autism spectrum disorder	
H2-CBD	Inflammation	Ben-Shabat et al., 2006 [18]
H4-CBD	Inflammation	Ben-Shabat et al., 2006 [18]
HU-446	Inflammation	Kozela et al., 2016 [19]
HU-465	Inflammation	Kozela et al., 2016 [19]
DMH-CBD	Inflammation Cancer Pain Neuroprotection	Burstein et al., 2015 [11] Juknat et al., 2016 [20]
HU-330	Inflammation	Sumariwalla et al., 2004 [21]
	Immunosuppresion	
HU-410	Inflammation	Mechoulam et al., 2008 [22]
HU-427	Inflammation	Mechoulam et al., 2008 [22]
HU-432	Inflammation	Mechoulam et al., 2008 [22]
HU-331	Cancer	Kogan et al., 2003 [23]
VCE-004.8/EHP-101	Inflammation Fibrosis Neuroprotection Remyelination	Del Rio et al., 2016 [24] Navarrete et al., 2018 [25] García-Martin et al., 2018 [26] García-Martin et al., 2019 [27] Navarrete et al., 2020 [28]

Now, due to its beneficial properties, the business surrounding the use of CBD in different products is increasing. In addition, CBD scaffolds have attracted increasing consideration for medicinal chemists. Therefore, CBD constitutes one of the most studied cannabinoids in neurodegenerative and demyelinating diseases where CBD has shown benefits in preclinical studies, warranting further investigation.

## 2. Cannabidiol: General Pharmacology and Therapeutic Profile

The understanding of cannabinoid pharmacology is continuously increasing, and the therapeutic effects of agonists and antagonists of the cannabinoid receptors type 1 and 2 (CB_1_R and CB_2_R) have been proposed for the treatment of several human disorders. This has been the result of several preclinical and clinical observations in which interactions with the cannabinoid receptors seem to alter molecular pathways that are responsible for the development of the diseases [29]. CBD is a potential candidate for clinical use thanks to its notable lack of psychotropic action and to its remarkable tolerability profile in humans [30].

CBD was identified by Adams et al. at the University of Illinois in 1940 but its structure was not completely clarified until the 1960s by Mechoulam et al. [3]. Up until now, the mechanisms of action of CBD are not totally known. It has been determined that CBD modulates central nervous system (CNS) receptors such as CB_1_R (negative allosteric modulator), CB_2_R, peroxisome proliferator-activated receptor-gamma (PPARγ), serotonin 1A receptor (5-HT1A), transient receptor potential cation channel subfamily V member 1 (TRPV1) and G protein-coupled receptor 55 (GPR55). CBD may antagonize CB_1_R receptor function by negative allosteric modulation of the orthosteric receptor site [31]. Regarding CB_2_R receptor, although CBD is a weak agonist of this receptor [32] it has been described that its activation could provide an anti-inflammatory and anti-oxidative effects [33]. Furthermore, CBD may act as an inverse agonist that could explain in part its anti-inflammatory properties inhibiting immune cell migration [34,35]. In vitro assays have shown that CBD is a weak agonist of PPARγ but in vivo assays demonstrated that some CBD biological activities can be blocked by pharmacological inhibition of PPARγ, suggesting that some metabolites of CBD may account for its activity of this nuclear receptor [36]. Furthermore, it has been described that CBD causes analgesia in a TRPV1-dependent manner and ameliorates anxiety through 5-HT1A receptor [37]. Besides, 5-HT1A receptor activation is also involved in CBD neuroprotection in in vitro adult and rat newborn models of the acute hypoxic-ischemic brain [38]. Likewise, CBD has been described as functional antagonist of the GPR55 receptor that can be relevant to explain the anticonvulsant activity of CBD [39].

Clearly, the impact of CBD on the provides many health benefits. Unfortunately, most of this evidence to date comes from animal studies and anecdotal human experience, since very few well-controlled human studies have been conducted with CBD, although this tendency is changing.

### The Endocannabinoid System

Due to the interest in recreational and medical uses of marijuana, efforts were made early in the sixties to identify the major cannabinoids in the cannabis plant [40,41]. These attempts resulted in the discovery of Δ9-THC, CBD, and cannabinol (a processing product of Δ9-THC) [3,42,43]. Then, using a synthetic radiolabeled Δ9-THC analogue, high-affinity binding sites for Δ9-THC in the brain were discovered as the CB_1_R [44], a G protein-coupled receptor (GPCR). Afterwards, a second G protein-coupled receptor (GPCR) called CB_2_R was identified outside the CNS, mainly in the immune system [45]. As a result, the cloning and identification of the two main cannabinoid receptors led to isolation of endogenous CB_1_R and CB_2_R ligands and the discovery of the endocannabinoid system (ECS). The lipids anandamide (N-arachidonoylethanolamide or AEA) and 2-arachidonoylglycerol (2-AG), identified in the brain and intestinal tissues, were shown to activate both receptors with high affinity and as consequence these lipids were named endocannabinoids. The levels of these endocannabinoids is regulated by enzymes, including fatty acid amide hydrolase (FAAH) [46] and monoacylglycerol lipase (MAGL) [47], that metabolize AEA and 2-AG, respectively. To partly explain their multipronged bioactivities, exogenous and endogenous cannabinoids also interact with non-cannabinoid receptors as described above for CBD [48].

Modifications in the ECS are frequently observed in neurological diseases [49] and genetic and pharmacological changes of this system in animal models suggest a major role for this system in neurodegenerative disorders and demyelinating diseases [50,51]. The ECS is a complex system due to the promiscuity of mediators and its interactions with other metabolic pathways. The regulation of the ECS components alters the endocannabinoid-related system, known as the endocannabidiome (eCBome). This complicated system presents a challenge for the discovery of novel bioactive molecules inspired in endocannabinoid and also suggests new chances for the utilization of non-psychotropic cannabinoids such as CBD and derivatives of CBD, which frequently modulate several eCBome proteins. In addition, lifestyle, including the lipid dietary component, habits, and environment are suggested to have an impact on the eCBome, which seems to be relevant in many physiological and pathological conditions [29,52].

## 3. Demyelinating Diseases

**Figure 1:**
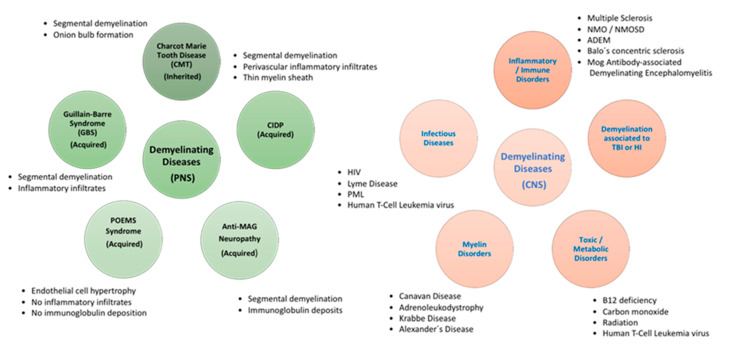
Demyelinating diseases.

Myelin is believed to be generated in early gnathostomes by a glial precursor, which later produce the different Schwann cell (SC) and oligodendrocyte lineages [53,54]. In fact, the global organization of myelinated axons is similar in the central nervous system (CNS) and peripheral nervous system (PNS), regarding their functions in saltatory transmission. However, Schwann cells and oligodendrocytes present considerable variations in the development and formation of myelin. In line with this, demyelinating diseases are limited to those involving PNS myelinated fibers or CNS fibers (Figure 1).

The myelin disorders can be categorized into several categories according to their etiologies: demyelination associated to inflammation, demyelination associated to virus, loss of myelin produced by metabolic imbalances, loss of myelin due to hypoxic-ischemic conditions and demyelination caused by brain injury. Most of the different categories overlap in pathogenesis but this organization may be helpful to establish a diagnosis. The prognosis of these diseases is generally difficult, and no curative treatment is currently available.

Several diseases involving significant injury to axons and glial cells, especially SC in the PNS, are classified as peripheral demyelinating diseases (PDD) [55]. Schwann cells, which are derived from the neural crest, represent the main glial cells in peripheral nerves. The development of SC happens through different embryonic and postnatal periods, which are strictly controlled by several cellular signaling pathways. Initially, the undifferentiated SC matures into either myelinating or non-myelinating SC and covers around axons, thus constituting the process named myelination [56]. The myelin sheath is composed of various coats of lipids and lipoprotein plasma membranes of SC which are arranged around the axon of neurons [57]. In PNS, the demyelination process involves the damage of the myelin sheath due to the injury on SC [58]. Currently, there are no consistent biomarkers for PNS-associated disorders and the diagnosis is based on several studies such as electrophysiological and cerebrospinal fluid (CSF) analysis. PDD can be classified in two main groups: Acquired Demyelinating Diseases and Inherited Demyelinating Diseases (Figure 1).

The first group comprises four main type of disorders such as Guillain-Barre Syndrome (GBS), chronic inflammatory demyelinating polyradiculoneuropathy (CIDP), anti-myelin associated glycoprotein (MAG) neuropathy and polyneuropathy, organomegaly, endocrinopathy, m protein and skin changes (POEMS) syndrome. GBS is a severe idiopathic autoimmune demyelinating disease associated to acute ascending neuromuscular palsy [59]. A high percentage of GBS cases have been related to autoantibodies related with several bacterial and viral infections [60,61,62]. Emerging information suggests that acute respiratory syndrome coronavirus-2 (SARS-CoV-2 or COVID-19) can cause GBS and several neurological autoimmunity-related diseases requiring attention for quick diagnostic and treatment [63]. MAG neuropathy is caused by circulating monoclonal antibodies towards the human natural killer-1 epitope. This epitope is expressed on adhesion molecules present in peripheral nerves such as the glycoprotein MAG. A low expression of MAG affects the myelin sheath structure and axonal function. This progressive disease causes mild to moderate distal muscle fragility, with gradual sensory ataxia and recurrent tremors [64]. CIDP is associated with a gradual loss of sensorimotor functions [65]. There are several effective treatments based on immunoglobulin, corticosteroids, and plasma exchange treatments, but long-lasting treatments are needed. Regarding POEMS syndrome, this is an unusual paraneoplastic syndrome with demyelinating neuropathy produced by a disorder related to plasma cell proliferation. The association of vascular endothelial growth factor (VEGF) with POEMS syndrome is very effective in clinical diagnosis as accurate biomarker and monitoring responses to treatment. POEMS patients present high serum level of VEGF, although low levels are described upon effective treatment [66].

The second group of hereditary demyelinating diseases include Charcot Marie tooth disease (CMT). Although CMT is an uncommon inherited neurological disease, it is the major disorder that affects the peripheral nerves. CMT patients, despite their genetic heterogeneity, typically present an indolent, length-dependent, sensorimotor polyneuropathy [67].

Currently, synthetic drugs and natural products are used for the management of PDD. Nevertheless, these diseases remain misdiagnosed due to the absence of solid biomarkers and disease safe-diagnostic criteria. Therefore, the search for new therapies and accurate biomarkers are essential to address this type of neuropathic disease [68].

Demyelinating disorders of the CNS have different etiologies and are divided into primary, such as MS and other idiopathic inflammatory-demyelinating diseases (IIDDs), and secondary, such as infective, ischemic, metabolic, or toxic diseases (Figure 1). These CNS demyelinating diseases comprise MS and its acute variant Marburg disease, also neuromyelitis optica (NMO), Balo’s concentric sclerosis, acute disseminated encephalomyelitis (ADEM), and ADEM’s hyperacute variant, acute hemorrhagic leukoencephalitis (AHL) (Figure 1). The term IIDD includes several CNS disorders that are classified according to their severity, clinical progression, and lesioned zone, as well as their pathological outcomes. The spectrum of diseases involves monophasic, multiphasic, and progressive disorders. Aggressive types of IIDD include a plethora of disorders that share the symptomatology, an acute clinical course, and atypical outcomes on magnetic resonance imaging (MRI). Marburg disease is the classic fulminant IIDD, but it is extremely rare. Baló’s concentric sclerosis, which is considered a variant of MS and ADEM, can also appear with acute attacks [69].

MS is characterized by inflammation-related injury, principally to myelin structure and composition from nerves in the brain (including optic nerves) and spinal cord, causing axonal damage and neurodegeneration. The most frequent forms of MS are the relapsing-remitting (RR) and secondary progressive (SP) forms, although it can also present a progression from onset (primary progressive (PP). The presentation of demyelinating lesions distributed in time and space are critical in the clinical diagnosis of MS. In addition to the neurological symptoms, lesions consistent with MS determined by MRI, along with presentation of oligoclonal bands in CSF and findings of abnormal visual evoked potentials, are proposed to provide an accurate diagnosis [70]. Neuromyelitis optica spectrum disorder (NMOSD) and myelin oligodendrocyte glycoprotein (MOG) antibody (Ab)-associated disease are also inflammatory CNS demyelinating disorders although clinically and pathologically they differ from MS and are far less common. NMO has been identified as a disease distinct from MS due to the identification of an NMO-specific autoantibody directed against aquaporin-4 (AQP4-Ab), the major water channel in the CNS [71].

Among the infectious inflammatory demyelinating disorders, progressive multifocal leukoencephalopathy (PML) is an aggressive CNS infection caused by JC virus (JCV). The disease is triggered by a JCV that selectively damages the oligodendrocytes, causing demyelination. Therapy with monoclonal antibody treatment or other immunomodulatory drugs, generally applied to MS patients, has also been used for PML treatment [72].

Traumatic brain injury (TBI) and stroke are major pathologies which result in demyelination. The neurovascular unit (NVU) is constituted by neurons, endothelial cells, smooth muscle cells, pericytes, astrocytes, and microglia [73]. This NVU is affected by secondary injuries after TBI and suffer alterations such as a reduction of blood–brain perfusion with adverse effects on the correct function of the neurons [74]. During the past decades, cerebrovascular dysfunction has been associated with a poor prognostic outcome. In addition, alterations in the structure of the blood-brain barrier (BBB) trigger edema generation with interference in the brain homeostasis. This alteration exacerbates the secondary injury processes including excitotoxicity and inflammation. For these reasons, TBI has been considered a chronic brain disease with molecular alterations in the BBB after the initial injury [75]. This long-term alteration of the blood-brain barrier could be responsible of premature aging of the brain after TBI [75,76]. Research on animal models have shown that cannabinoids targeting CB_2_R after TBI improve neurobehavioral manifestations and memory tests and the neurological insufficiency, and diminish motor deficit through downregulation of proinflammatory markers. Furthermore, the modulation of cannabinoid system reduces oedema generation and BBB permeability, avoiding neuronal cell death and upregulating the levels of adherence junction proteins (reviewed in [77]). Moreover, PPARγ seems to be another interesting target to prevent neuroinflammation and demyelination in TBI [78].

At present, advancement has been made in identifying the pathogenesis of demyelinating disorders, but we have to discover their origin or a therapeutic treatment for these debilitating diseases that affect millions of young adults around the world. The development of new therapies for the treatment of these diseases remains a challenge. Indeed, the support of the beneficial potential of cannabinoids, especially CBD, for the control of pathological events related to these diseases is increasing.

## 4. Cannabidiol and Demyelinating Diseases

During the past decade, the therapeutic potential of cannabinoids for treating demyelinating diseases, specifically MS, has been well-studied. It is now established that CBD and various CBD-derivatives confer neuroprotective effects and attenuate the inflammatory process in several demyelinating animal models [19,20,25,79]. For instance, the impact of CBD has been determined in hypoxic-ischemic immature brain. CBD, at micromolar concentrations, reduces the levels of inflammatory markers such as interleukin-6 (IL-6), tumor necrosis factor-α (TNF-α), cyclooxygenase-2 (COX-2), and inducible nitric oxide synthase (iNOS), through activation of CB_2_R and A2A receptors [80,81]. Regarding A2A receptors, it has been described that CBD increases adenosine signaling inhibiting adenosine uptake [82].

Currently the search for new remyelinating therapies is focused principally on identifying the factors that promote repair of the myelin sheath. Demyelinating diseases share as a common feature the principal pathogenic process that targets the myelin sheath. This neuronal covering allows proper conduction of the nerve impulses. MS is characterized in particular by a decrease in the number of oligodendrocytes producing myelin in the CNS and by progressive axonal deterioration. As it occurs in most demyelinating diseases, damage of the myelin sheath triggers regeneration mechanisms, a process named remyelination. Oligodendrocytes responsible for production of mature myelin are derived during postnatal development from immature cells named oligodendrocyte progenitor cells (OPCs). OPCs stay in the adult brain and produce new mature oligodendrocytes when the myelin is injured. The beneficial role of CBD against injury to OPCs mediated by the immune system has been described. Indeed, cells treated with CBD present less oxidative stress avoiding the generation of reactive oxygen species. Furthermore, the treatment of OPC with CBD prevented apoptosis by mechanisms independent from CB_1_R, CB_2_R, TRPV1 or PPARγ receptors [13]. OPCs have remarkable metabolic conditions during development because they can differentiate to generate myelin segments, indicating they need a satisfactory blood supply. However, processes that coordinate myelination and angiogenesis must be better defined. It has been described that Hypoxia-Inducible Factor (HIF) is an important regulator of postnatal myelination. HIF-1α activation may play a role in the inflammatory and the remitting phases of MS [83]. Moreover, activation of the HIF pathway may also be associated to neuronal protection and remyelination [84]. Thus, we have described that VCE-004.8, a synthetic aminoquinone derivative of cannabidiol, could be a potential drug to treat MS by regulating the immune response and supporting neuroprotection and axonal regeneration through activation of the hypoxia-inducible factor pathway. Furthermore, this novel synthetic cannabinoid, which also acts as a dual PPARγ and CB_2_R agonist, presents potent anti-inflammatory activity [25].

Neonatal hypoxia-ischemia (HI), which causes myelination disorders and is related to cerebral palsy, presents a complex pathophysiology which includes oxidative stress, excitotoxicity, and severe inflammatory response [85,86]. Due to this altered environment, oligodendrocyte progenitors are particularly sensitive. Thus, OPC injury is increased in the brain, both in animal models and human newborns after HI damage [87] which eventually leads to hypomyelination [88]. The protective role of CBD after HI injury in newborn animals has been described in neuronal and glial cells [89].

MS, as well as other demyelinating diseases, presents many of the characteristics of autoimmune disorder with rupture of the BBB. The BBB is an extraordinary composition of endothelial cells, pericytes, which are enclosed and supported by astrocytes and perivascular macrophages. In pathological circumstances, peripheral lymphocytes are activated and infiltrate the CNS to trigger an immune response injuring myelin and axons. In an experimental model of MS, the Theiler’s murine encephalomyelitis virus-induced demyelinating disease, it has been demonstrated that CBD ameliorates the symptomatology of the disease. It has been shown that intraperitoneal treatment with CBD reduces the extravasation of leukocytes from the systemic circulation by downregulating the expression of several chemokines as well as by decreasing microglia activation [90]. Furthermore, the effect of CBD on BBB permeability has been determined by using human brain microvascular endothelial cells and human astrocyte co-cultures as a BBB model. In this model, CBD restored the BBB permeability produced by oxygen-glucose deprivation (OGD). Treatment was more efficient when it was administered prior to OGD, but positive results were detected up to two hours into reperfusion. The protective effect was dependent on PPARγ and relatively reduced by a 5-HT1A receptor antagonist but was independent of CB_1_R, CB_2_R, TRPV1 or Adenosine A2A receptors [91].

During the past few decades, our understanding about the loss of myelin after stroke and TBI has increased. Previous research on these pathologies highlighted changes in neuronal cells within the gray matter. Recently, several studies have shown the same importance of the white matter integrity in long-term recovery. Demyelination following brain injury causes long-term sensory, motor, and cognitive insufficiencies due to the adult brain’s low capability to regenerate oligodendrocyte cells and the restoration of axonal myelin. New molecules that control the process of remyelination may offer novel therapies to restore white matter integrity and improve long-term neurological improvement in stroke and TBI patients. In addition, it has been shown that treatment with oral CBD oil restored behavioral dysfunctions and normalized the cortical biochemical changes associated with TBI. Therefore, CBD has been proposed as a pharmacological tool to improve neurological dysfunctions triggered by the trauma [92]. Furthermore, several studies have shown that short-term treatment with CBD in a mouse model of brain injury, such as bilateral common carotid artery occlusion (BCCAO), is capable of improving motor and cognitive disability by activating a complex mechanism related with an increase in hippocampal levels of brain-derived neurotrophic factor (BDNF) and microtubule-associated protein 2 (MAP-2) proteins, resulting in stimulation of neurogenesis. Because of these effects, the treatment with CBD ameliorated neuroinflammation and neuronal death in the hippocampal zone [9,93].

## 5. Medicinal Chemistry of Synthetic and Natural Derivatives of Cannabidiol

Of the over one hundred phytocannabinoids discovered in *Cannabis sativa*, seven have been categorized as CBD-type compounds, including CBD [94,95]. All of them present the same configuration as CBD. Among these natural analogs, cannabidiolic acid (CBDA) and cannabidivarinic acid (CBDVA-C3), which are C30-carboxylic derivatives, have been isolated. Moreover, cannabidiorcol (CBD-C1), cannabidiol-C4 and cannabidivarin (CBDV), which vary from CBD by the length of their C40-side chain, have been identified. Finally, cannabidiol monomethyl ether (CBDM), the C60-methoxy CBD analog, has also been isolated from the plant. Although these natural CBD derivatives present potential therapeutic benefits (Table 1), only a few pharmacological studies have been described [14,15,16,17].

Because of the favorable therapeutic benefits of CBD in a variety of diseases, synthetic CBD derivatives have also been taken into consideration by drug discovery projects, with the purpose of improving the potency, efficacy, and/or pharmacokinetic properties of this natural cannabinoid. To obtain new synthetic analogs, a series of structural modifications such as hydrogenation of CBD produced the dihydro and tetrahydrocannabidiol derivatives H2-CBD and H4-CBD [18]. These molecules have been attributed anti-inflammatory properties because of their effects on the generation of reactive oxygen species, nitric oxide, and tumor necrosis factor. Furthermore, the hydroxy-CBD enantiomers, named HU-446 and HU-465, have shown potential anti-inflammatory effects in a proinflammatory model of encephalitogenic T cells (Table 1). Specifically, both HU-446 and HU-465 prevented the production of IL-17, a crucial autoimmune cytokine, from MOG_35-55_-stimulated T(MOG) cells. These data indicated that both CBD derivatives have anti-inflammatory effects in autoimmune diseases [19].

The synthesis of dimethylheptyl (DMH) CBD derivatives such as DMH-CBD, HU-320 [21], and 7-OH-DMH-CBD have been described by Mechoulam and colleagues [96]. In the case of DMH-CBD, it was reported that this derivative abolishes the production of proinflammatory cytokines and prevents microglia reactivation by generating an adaptive cellular response, thus avoiding inflammation and oxidative injury (Table 1). In addition, DMH-CBD reduced the proliferation of pathogenic activated TMOG cells [20]. For such derivative, remarkable benefits such as anti-inflammatory, analgesic, neuroprotective or antitumor effects have been described, and it has been used as a pharmacological tool in many cannabinoid studies supporting the progress in this field [11].

Finally, other interesting modifications have consisted of changes in the C40-alkyl chain with the purpose of improving oral bioavailability, modifications of the resorcinol hydroxyl groups, thus generating new molecules (named HU-410, HU-427, and HU-432) that present anti-inflammatory activities as reported in the patent literature (Table 1) [22]. The development of new quinone derivatives of CBD has also been investigated.

The first quinone derivative of CBD, named HU331, was described by Mechoulam et al. by oxidation of CBD [97] and its antineoplastic activity was reported (Table 1) [23]. Quinone-based drugs causing anti-infective and antitumoral effects are frequently applied in clinical practice, but their use for chronic therapies is not recommended due to their reactivity and toxicity. HU-331 is a thiol-trapping compound that generates reactive oxygen species (ROS), affects the mitochondria transmembrane potential and causes cytotoxicity in primary and transformed cells in vitro [98].

We have generated a non-thiophilic and chemically stable derivative of a CBD aminoquinone (VCE-004.8) that acts as a dual agonist of PPARγ and CB_2_R. VCE-004.8 does not have affinity for the CB_1_R receptor and presents potent antifibrotic activity in vitro and in vivo (Table 1) [24]. Furthermore, we have described that VCE-004.8 also activates the HIF pathway. In fact, we have reported that VCE-004.8 stabilizes and activates HIF-1α and HIF-2α in human microvascular endothelial cells, oligodendrocytes, and microglia cells [25]. VCE-004.8 ameliorated neuroinflammation and prevented myelin loss in several murine models of MS, such as Experimental autoimmune encephalomyelitis (EAE) and Theiler’s virus-induced demyelinating disease [25]. Recently, we have reported that EHP-101, which is an oral lipidic formulation of VCE-004.8, also had efficacy in EAE and induced remyelination in two demyelination models induced by cuprizone. [28]. Hence, EHP-101 could be a promising cannabinoid-derived drug candidate for the treatment of different forms of MS. In addition, EHP-101 also demonstrated efficacy in a murine model of systemic sclerosis (SSc) [26,27]. EHP-101 is now under evaluation in a Phase II study in SSc patients (ClinicalTrials.gov: NCT04166552) and the initiation of a Phase II study in MS patients is underway.

## 6. Clinical Trials of Cannabidiol Focused on Demyelinating Disorders

Currently, medicine may be focused on CBD as a new treatment for patients with reduced conventional options and medical professionals are often asked about CBD products by patients, family, and patient associations. Due to its minimal toxicity in humans, an interesting number of trials have been performed to determine the clinical efficacy of CBD in different pathologies. Numerous CBD formulations have been assessed in preclinical studies for various pharmaceutical properties, such as anti-nausea, anti-emetic, anti-tumor, anti-inflammatory, antidepressant, anti-psychotic, and anti-anxiolytic [10,11,12,99] benefits. Nevertheless, the variation in CBD quality, the type of drug formulations applied, and the minimal sample sizes compromise the development of these preclinical studies.

As we have previously indicated in this review, to date the FDA has approved three CBD- and Δ9-THC-based medicines. Dronabinol (Marinol, Syndros) which is a synthetic form of THC in an oily base, administered to stimulate appetite in AIDS patients and for the improvement of nausea and vomiting associated with cancer chemotherapy. Nabilone (Cesamet) is another Δ9-THC analog for the treatment of nausea in patients undergoing chemotherapy. Cannabidiol oral solution (Epidiolex) is authorized in the USA as therapy of two severe rare childhood epilepsy disorders (Dravet syndrome and Lennox-Gastault syndrome). A fourth medication, nabiximols (Sativex), a combination of Δ9-THC and CBD, is sold legally in more than ten countries including Canada, Mexico, and parts of Europe, for the treatment of muscle spasticity and neuropathic pain in multiple sclerosis, and the FDA recently recognized an Investigational New Drug application for nabiximols. This aromatized water-ethanol oral-mucosal spray was created to offer a simple delivery system. Specifically, this method of dispensation allows rapid entry to the circulation through the oral mucosa with an extremely rapid plateau of plasma concentration, preventing the complications of the gastrointestinal route. Furthermore, it has been shown that co-administration of CBD to Δ9-THC counteracts the undesirable effects of Δ9-THC alone [100].

Until now there are about 2,500 clinical trials focused on demyelinating disorders and among them only 30 studies have been related to the benefits of cannabis or cannabinoids. In fact, there are 19 clinical trials that address the effect of CBD in demyelinating disorders, specifically in MS. Medicinal cannabis has been researched as potential therapy for multiple sclerosis symptomatology, such as pain and spasticity [101]. During the first 10 years of initial MS diagnosis, up to 80 percent of patients are affected by moderate spasticity and the numbers of affected patients rises over time [102,103]. The study called “*The Cannabinoids for Treatment of Spasticity and Other Symptoms Related to Multiple Sclerosis (CAMS)*” was a major randomized trial that explored spasticity in more than 600 MS patients. This study did not observe changes in the Ashworth Spasticity Scale between either oral cannabis extract contrasted with placebo after 15 weeks. However, objective improvement in mobility and pain suggested cannabinoids might be clinically beneficial [104].

Many studies have investigated the pharmacological properties of cannabinoids in demyelinating diseases such as MS. In fact, most of the trials have been executed using Sativex which is suggested as a second line therapy for spasticity in MS patients who do not respond to other anti-spasticity treatment and who experienced clinically remarkable improvement in symptoms associated to spasticity during the onset of the trial. Since the first reported study in 2003, when an initial controlled study determined that cannabis extracts could improve intractable neurogenic symptoms [105,106], and during the last decade, several clinical trials have evaluated the efficacy of Sativex as a supplementary treatment for symptomatology recovery in patients with MS-related spasticity and neuropathic pain.

In 2010, a meta-analysis of the effectiveness and security of nabiximols on spasticity in 666 MS patients showed remarkable superior percentage of treated patients as responders and treated patients also reported remarkable improvements [107]. In the same year, a double-blind, randomized, placebo-controlled, parallel-group study of nabiximols was performed in subjects with symptoms of spasticity due to MS. This randomized controlled trial studied the treatment effects in 337 subjects for 15 weeks and no important improvement in the mean spasticity numerical rate scale (NRS) was observed in intent-to-treat (ITT) analysis but responder per protocol (PP) analyses confirmed that nabiximols treatment caused an important decrease in treatment-resistant spasticity, in patients with progressive MS and severe spasticity [108]. Later, Novotna et al., published the results derived from a randomized, double-blind, placebo-controlled, parallel-group, enriched-design study of nabiximols, as add-on therapy, in MS subjects with refractory spasticity. In this trial, 241 randomized patients received treatment with nabiximols, as add-on therapy, in a single-blind manner for 28 days, after which those showing positive effects in spasticity of ≥ 20% progressed to 84-days randomized, placebo-controlled phase. ITT analysis showed an important significant variation positive for nabiximols treatment [109]. In 2014, the results derived from the first Phase III placebo-controlled study of the efficacy of the nabiximols to improve central neuropathic pain (CNP), which occurs in many MS patients, were published. More than 300 subjects were randomized to Phase A (167 received nabaximols and 172 received placebo). Of those who finished Phase A, 58 started the randomized-withdrawal phase. The results of this research were ambiguous, with contradictory conclusions in the study [110]. In 2014, a multicenter, non-interventional study called MOVE-2 showed the effect of nabiximols in 276 patients [111]. After one month, nabiximols improved resistant MS spasticity (MSS) in 74.6% of patients. After three months, 55.3% of subjects had persisted to receive nabiximols and the mean NRS score had decreased by 25% from baseline. This study MOVE-2 was prolongated for 12 months and 52 patients were incorporated to the effectiveness analysis. The mean spasticity NRS reduced considerably from 6.0 ± 1.8 at baseline to 4.8 ± 1.9 after the first 30 days and remained on 4.5 ± 2.0 after 12 months [112]. These data confirmed the long-term efficacy and tolerability of nabiximols for the therapy of resistant MSS in clinical.

Recently, Marinelli et al. proposed a novel study to identify if nabiximols could be useful ameliorating spasticity in stroke and to investigate its tolerability and security by accurate cardiovascular monitoring. The study will recruit 50 patients with spasticity following stroke to be dosed with nabiximols in a double-blind placebo-controlled cross-over study [113]. Finally, in 2019 the results derived from the study named SAVANT were published, evaluating the effects of nabiximols as add-on therapy versus optimized first line antispastics in resistant MS spasticity. In this double-blind, placebo-controlled randomized clinical trial of 191 patients who entered Phase A, 106 were randomized in Phase B to receive add-on nabiximols spray (*n* = 53) or placebo (*n* = 53). The percentage of clinically relevant responders after 12 weeks was significantly superior with nabiximols than placebo [114].

At present, data indicates that cannabidiol and some derivatives have a remarkable role in the modulation of myelinating processes, and it has been suggested as a promising approach in the treatment of demyelinating diseases. Although serious advances are being made in the development of new cannabidiol derivative drugs and therapeutic targets, the collaboration of researchers and pharmaceutical companies is needed to achieve successful outcomes.

## Data Availability

Not applicable.

## Abbreviations

2-AG	2-arachidonoylglycerol
ADEM	Acute Disseminated Encephalomyelitis
AEA	Anandamide
AHL	Acute Hemorrhagic Leukoencephalitis
AIDS	Acquired Immunodeficiency Syndrome
BBB	Blood-Brain Barrier
BCCAO	Bilateral Common Carotid Artery Occlusion
BDNF	Brain-Derived Neurotrophic Factor
CB1R	Cannabinoid receptor type 1
CB2R	Cannabinoid receptor type 2
CBD	Cannabidiol
CBDA	Cannabidiolic Acid
CBDM	Cannabidiol Monomethyl Ether
CBDV	Cannabidivarin
CBDVA	Cannabidivarinic Acid
CIDP	Chronic Inflammatory Demyelinating Polyradiculoneuropathy
CMT	Charcot Marie Tooth Disease
CNP	Central Neuropathic Pain
CNS	Central Nervous System
COX-2	Cyclooxygenase-2
CSF	Cerebrospinal Fluid
EAE	Experimental Autoimmune Encephalomyelitis
eCBome	Endocannabidiome
ECS	Endocannabinoid System
FAAH	Fatty Acid Amide Hydrolase
FDA	Food and Drug Administration
GBS	Guillain-Barre Syndrome
GPCR	G protein-coupled receptor
GPR55	G protein-coupled receptor 55
HI	Hypoxia-Ischemia
HIF	Hypoxia-Inducible Factor
IIDDs	Idiopathic Inflammatory-Demyelinating Diseases
IL-6	Interleukin-6
iNOS	Inducible Nitric Oxide Synthase
MAG	Anti-Myelin Associated Glycoprotein
MAGL	Monoacylglycerol Lipase
MAP-2	Microtubule-Associated Protein 2
MOG	Myelin Oligodendrocyte Glycoprotein
MRI	Magnetic Resonance Imaging
MS	Multiple Sclerosis
NMO	Neuromyelitis Optica
NMOSD	Neuromyelitis Optica Spectrum Disorder
NRS	Numerical Rate Scale
NVU	Neurovascular Unit
OGD	Oxygen-Glucose Deprivation
OPCs	Oligodendrocyte Progenitor Cells
PDD	Peripheral Demyelinating Diseases
PML	Progressive Multifocal Leukoencephalopathy
PNS	Peripheral Nervous System
POEMS	Polyneuropathy, Organomegaly, Endocrinopathy, M protein and Skin changes Syndrome
PP	Primary Progressive
PPARγ	Peroxisome Proliferator-Activated Receptor-Gamma
ROS	Reactive Oxygen Species
RR	Relapsing-Remitting
SC	Schwann Cell
SP	Secondary Progressive
TBI	Traumatic Brain Injury
TNF-α	Tumor Necrosis Factor-α
TRPV1	Transient Receptor Potential Cation Channel Subfamily V Member 1
VEGF	Vascular Endothelial Growth Factor
Δ9-THC	Δ9-tetrahydrocannabinol
